# Esophageal perforation during or after conformal radiotherapy for esophageal carcinoma

**DOI:** 10.1093/jrr/rru031

**Published:** 2014-06-08

**Authors:** Hai-yan Chen, Xiu-mei Ma, Ming Ye, Yan-li Hou, Hua-Ying Xie, Yong-rui Bai

**Affiliations:** Department of Radiation Oncology, Ren Ji Hospital, School of Medicine, Shanghai Jiao Tong University, Shanghai 200127, China

**Keywords:** esophageal carcinoma, radiotherapy, esophageal perforation, extracapsular lymph node involvement, covered self-expandable metallic stent

## Abstract

The aim of this study was to analyze the risk factors and prognosis for patients with esophageal perforation occurring during or after radiotherapy for esophageal carcinoma. We retrospectively analyzed 322 patients with esophageal carcinoma. These patients received radiotherapy for unresectable esophageal tumors, residual tumors after operation, or local recurrence. Of these, 12 had radiotherapy to the esophagus before being admitted, 68 patients had concurrent chemoradiotherapy (CRT), and 18 patients had esophageal perforation after RT (5.8%). Covered self-expandable metallic stents were placed in 11 patients. Two patients continued RT after stenting and control of infection; one of these suffered a new perforation, and the other had a massive hemorrhage. The median overall survival was 2 months (0–3 months) compared with 17 months in the non-perforation group. In univariate analysis, the Karnofsky performance status (KPS) being ≤70, age younger than 60, T4 stage, a second course of radiotherapy to the esophagus, extracapsular lymph nodes (LN) involving the esophagus, a total dose >100 Gy (biologically effective dose_−10_), and CRT were risk factors for perforation. In multivariate analysis, age younger than 60, extracapsular LN involving the esophagus, T4 stage, and a second course of radiotherapy to the esophagus were risk factors. In conclusion, patients with T4 stage, extracapsular LN involving the esophagus, and those receiving a second course of RT should be given particular care to avoid perforation. The prognosis after perforation was poor.

## INTRODUCTION

Esophageal carcinoma is a common cancer in developing countries; the overall 5-year survival has improved significantly over the past few decades but remains poor at 19% [1]. Radiation treatment plays a large role in the treatment of esophageal cancer, both as primary therapy for unresectable tumors and for local recurrence after surgery, which has been reported at 30% for radical resections and 60% for an R1 or R2 resection [2–3]. Perforation of the esophagus is a severe side-effect of esophageal radiation, but reports of perforation after RT are rare, and cases that have been reported often involve perforation after conformal radiotherapy (RT) combined with intracavitary RT [4]. The aim of this retrospective analysis of 322 patients who received palliative or radical RT for esophageal carcinoma was to access the risk factors and prognosis for esophageal perforation during or after RT.

## MATERIALS AND METHODS

### Patients

Between Jan 2007 and Aug 2011, a total of 322 patients with esophageal carcinoma received mediastinal RT in our department. These patients received RT for unresectable esophageal tumors, residual tumors after operation, or local recurrence, and at least part of the esophagus was inside the RT field. Among them, 106 received RT for local recurrence, the T distribution was T0 in 69, T2 in 10, T3 in 17, and T4 in 10; 79 of them had local lymph node metastasis. Of the 216 patients treated for the first time, the T distribution was T1 in 4, T2 in 32, T3 in 101, and T4 in 79; 124 of them had local lymph node metastasis. All the patients were informed and consented to treatment (details in Table 1).

**Table 1.** Unilateral analysis of 322 patients with esophageal cancer

	Non-perforation (*n* = 304)	Perforation (*n* = 18)	*P*-value	OR
Age				
<60	105	14	0.001	0.149 (0.048–0.46)
≥60	199	4		
KPS				
>70	287	13	.004	0.155 (0.049–0.48)
≤70	17	5		
Sex				
Male	248	18	.051	0.932
Female	56	0		
RT for the second time				
Yes	8	4	.000	14.2 (4.07–49.2)
No	295	14		
RT for recurrence				
No	203	13	1.000	0.769 (0.67–2.26)
Yes	101	5		
LN invading the esophagus				
Yes	45	11	.0000	8.19 (2.96–22.6)
No	259	7		
Concurrent chemoradiotherapy				
Yes	59	9	.005	4.136 (1.57–10.87)
No	245	9		
Total radiation dose				
<100 Gy	297	14	.0002	12.12 (3.16–42.16)
≥100 Gy	7	4		
(BED_−10_)				
T4 stage				
Yes	77	12	.011	3.67 (1.39–9.63)
No	226	6		

Of the 322, 12 patients had received RT before being admitted, and their RT in this analysis represents a second course of therapy. The median interval between the two radiation treatments was 62 months, ranging from 5–172 months. Of these 12 patients, 10 were diagnosed as having anastomotic recurrence, and the other two as having supraclavicular lymph node (LN) metastasis. These patients were not suitable for surgery because of various medical reasons. The mean total dose was 126.9 Gy (93.6–156 Gy), dose calculation based on BED_−10_ (biologically effective dose_−10_) BED_−10_ = nd (1+(d/α)/β), with α and β ratio of 10; details in Table 2).

**Table 2.** Data of 12 patients receiving re-irradiation with esophageal cancer

Sex/age	Surgery	Recurrence site	Previous dose	Re-irradiation dose	BED_−10_	Interval (months)	Perforation	Response
M/78	No	Esophagus	60 Gy	18 Gy	93.6 Gy	24	No	SD
M/59	Radical	Anastomoses	50 Gy	60 Gy	132 Gy	36	No	CR
F/78	No	Esophagus	66 Gy	50 Gy	139.2 Gy	93	No	SD
F/74	No	Esophagus	66 Gy	50 Gy	139.2 Gy	13	No	SD
M/62	No	Esophagus	70 Gy	50 Gy	144 Gy	68	No	PR
F/72	No	Esophagus	68 Gy	60 Gy	153.6 Gy	95	No	PR
M/62	No	Esophagus	70 Gy	60 Gy	156 Gy	172	No	CR
M/71	No	Esophagus	70 Gy	60 Gy	156 Gy	90	No	PR
M/48	Palliative	Supraclavicular lymph node	70 Gy	20 Gy	108 Gy	5	Yes	PR
M/57	Radical	Anastomoses and lymph node	56 Gy	38 Gy	112.8 Gy	108	Yes	PR
M/53	Radical	Supraclavicular lymph node	70 Gy	40 Gy	132 Gy	19	Yes	PR
M/51	Radical	Anastomoses and lymph node	50 Gy	60 Gy	132 Gy	18	Yes	PR

SD = stable disease, CR = complete response, PR = partial response.

Before RT, thoracic CT, barium meal examination and a gastroscopy was performed to exclude those with perforation and those at high risk of perforation (having ulcer or thin esophageal walls). All but five patients had had pathologic examination, and these five patients had severe dysphasia and were diagnosed as having esophageal cancer after clinical and radiographic examination. Of the cases with pathological diagnosis, 279 had squamous carcinoma, seven had adenocarcinoma, eight had undifferentiated carcinoma, and the remaining five had other types of cancer. LNs > 10 mm in the short axis (or 5–10 mm but markedly enhanced in contrast-enhanced CT) were identified as metastatic lymph nodes.

Assessment of esophageal perforation was determined by leakage on iodine examination, or if any of the following manifestations were shown on the CT scan: (i) air in the soft tissues of the mediastinum surrounding the esophagus, (ii) abscess cavities adjacent to the esophagus in either the pleural space or mediastinum, or (iii) actual communication of an air-filled esophagus with an adjacent mediastinal or paramediastinal air-fluid collection [5].

Extracapsular LN involvement (ECLNI) was deemed positive when irregular nodal boundaries, infiltration of adjacent fat planes, thickening of adjacent fascia, and apparent invasion of adjacent structures were present [6]. LNs infused with the esophagus were considered extracapsular LNs involving the esophagus.

### RT plan

The gross tumor volume (GTV) was defined as any disease identified in pretreatment staging procedures including CT, PET and endoscopy. The clinical target volume (CTV) was defined as the GTV plus inclusion of the regional draining lymphatics (based on the primary tumor location). The radiation field encompassed the primary lesion as well as the regional LN drainage, which included the complete extent of visible tumor with a margin of 5 cm proximally and distally along the length of the esophagus and 1.5–2.5 cm otherwise around target tissues. Regional node drainage was defined by the primary lesion location. For mid-esophageal lesions, the adjacent middle and posterior mediastinal nodes were included. For distal esophageal or GE junction lesions, the lower para-esophageal, left gastric, and lesser curvature nodes were included. However, for patients who received a second course of RT, the planning target volume (PTV) was defined as the GTV plus a 0.5-cm margin in all directions.

Radiation was delivered by high-energy (6-MV) linear accelerators. All patients received 3D conformal RT, 78 of which had intensity-modulated RT (IMRT); patients receiving re-irradiation were all treated by IMRT. A total of 294 patients received 1.8–2.0 Gy per fraction per day, five days per week; 25 patients received late course accelerated RT, which was 2.0 Gy per fraction for 16 fractions, 2.2 Gy per fraction for 16 fractions, 2.3 Gy per fraction for five fractions, then 2.5 Gy per fraction for five fractions; another three patients received 1.5 Gy per fraction followed by 1.8–2.0 Gy per fraction for one day because of invasion of the tumor from the esophagus into the trachea.

### Concurrent chemoradiotherapy

Concurrent chemoradiotherapy (CRT) was administered to 69 patients, and the regimens included paclitaxal and cisplatin in 54 patients, docetaxel and cisplatin in six patients, 5-fluorouracil and cisplatin in three patients, capecitabine and oxaplatin in two patients, and a single agent of cisplatin in four patients.

### Statistical analysis

All statistical analyses were performed using the statistics program SPSS version 19.0 for Windows. Chi-square tests, Fisher exact analysis and *t* tests were used for univariate analysis, and logistic analysis was used for multivariate analysis.

## RESULTS

### Conditions of perforation

**Fig. 1.**
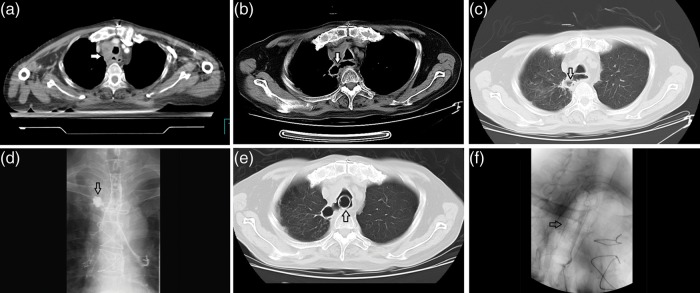
A patient with esophageal cancer who received radiotherapy after exploratory thoracotomy, (**a**) CT image when started the RT, the arrow head showed an extracapsular LN invading the esophagus, (**b**) CT image (mediastinal window) of the esophageal perforation (the arrow head indicates the actual communication of an air-filled esophagus with an adjacent mediastinal or paramediastinal air–fluid collection), (**c**) the perforation on lung window, (**d**) the leakage of iodine on iodine examination, (**e**) CT image after the placement of a self-expandable metallic stent (the arrow head indicates the stent), and (**f**) an X-ray after the placement of a stent (the arrow head indicates the stent).

Esophageal perforation was observed in 18 patients, i.e. an incidence rate of 5.6%. All of these patients had symptoms such as fever, cough or chest pain. After CT and iodine examination, a diagnosis of perforation was made accordingly (e.g. Fig. 1.)

Perforation occurred during RT in 10 patients, and 8–40 weeks after RT in eight patients; the mean and median intervals were 14 and 6 weeks, respectively.

All perforations were located at the level at which the esophagus was invaded by the tumor. The T stage distribution in the perforated group was T4 in 12 of the 18 patients. Of the six patients with non-T4 disease, five patients had extracapsular LNs that invaded the esophagus, and the other patients received a second course of RT. Extracapsular invasion of the esophagus from metastatic LNs in the perforated arm was observed in 11 patients. Based on the pre-RT and post-perforation CT examinations, a partial response was obtained in all of the 18 patients according to RECIST criteria, excluding the possibility of perforation due to tumor progression.

Perforated occurred during the first cycle of RT in 14 patients, and in the remaining four during re-irradiation. The mean radiation dose was 79.4 Gy (16.4–84 Gy) in the former group, and 118.2 Gy (96–132 Gy) in the latter group (calculated by BED_−10_) (details in Table 3).

**Table 3.** Data of 18 patients with esophageal perforation

Patient/sex/age	KPS	TNM stage	Re-irradiation	CRT^c^	ECLNI^d^
1/M/53	90	T4N1M1	No	No	Yes
2/M/41	80	T4N1M0	No	Yes	No
3/M/61	80	T4N0M1	No	Yes	No
4/M/60	90	T3N1M1	No	Yes	Yes
5/M/65	70	rT0N1M0	No	No	Yes
6/M/42	70	T4N0M0	No	Yes	No
7/M/56	90	T4N1M1^a^	No	No	Yes
8/M/63	60	T4N1M0	No	No	Yes
9/M/51	70	rT4N1M0	Yes^a^	Yes	Yes
10/M/57	90	rT2N1M0	Yes^b^	No	Yes
11/M/57	80	T3N1M0	No	Yes	Yes
12/M/53	80	rT0N1M0	No	Yes	Yes
13/M/48	80	rT3N1M0	Yes	No	No
14/M/50	60	T3N0M0	Yes	Yes	Yes
15/M/51	70	T4N1M1^a^	No	Yes	No
16/M/56	70	T4N1M1^a^	No	No	No
17/M/42	60	T4N1M1	No	No	Yes
18/M/56	90	T4N0M0	No	No	No

^a^Yes: the patient received mediastinum RT one year before re-irradiation for LN metastasis and anastomotic recurrence.

^b^Yes: the patient received mediastinum RT 108 months before re-irradiation for LN metastasis and anastomotic recurrence.

^c^CRT: concurrent chemoradiotherapy.

^d^ECLNI: extracapsular lymph nodes invading the esophagus.

### Result of re-irradiation

Of the 12 patients who received re-irradiation, two had complete response, seven had partial response, and the remaining three had stable disease. The median overall survival was 5 months.

### Treatment after perforation

After perforation, the patient's basic management included cessation of RT and oral intake, initiation of parenteral nutrition, intravenous broad-spectrum antibiotic, and intravenous administration of proton pump inhibitors as well as fluids.

Of the 18 patients, 11 received placement of covered self-expandable metallic stents, three had placement of nasal–gastric tubes, another three had percutaneous gastrojejunostomy, and one patient died before treatment. Massive hemorrhage occurred in three patients after stenting in this study.

Two patients continued RT after control of thoracic infection; one of them had a new perforation at the edge of the stent one month after delivery of 78.59 Gy (BED_−10_), and the perforation was also located at the site of the tumor, extending from the extracapsular LN. This patient died of thoracic infection eventually; the other patient, who continued RT, died one month after delivery of 65.24 Gy (BED_−10_) as a result of massive hemorrhage.

All of the patients with perforation had died by the time of this analysis. The median overall survival (OS) was 2 months (0–3 months) compared with 17 months in the non-perforation group, with *P* < 0.05. The major cause of death was infection involving the mediastinum, lung or cervical soft tissue, and subsequent sepsis; massive hemorrhage was the second cause of death.

### Statistical analysis

The following covariates were examined in our statistical models: age, sex, KPS, pathology, T stage, whether the patient had surgery, previous chemotherapy, previous mediastinal RT, treatment for local recurrence, tumor in the esophagus, extracapsular LNs involving the esophagus, total radiation dose, concurrent CRT and BED_−10_. Patients with age younger than 60 years old, KPS ≤70, T4 stage, a second course of RT, extracapsular LNs involving the esophagus, CRT and a total dose >100 Gy (BED_−10_) were at higher risk of perforation in the univariate analysis (details in Table 1). Age less than 60, T4 stage, extracapsular LNs involving the esophagus, a second course of RT and higher BED_−10_ were risk factors in the multivariate analysis (details in Table 4).

**Table 4.** Multivariate analysis of 322 patients with esophageal cancer

	B	S.E.	Wals	df	Sig.	Exp (B)
Younger than 60 or not	−1.526	0.719	4.512	1	0.034	0.217
Re-irradiation or not	8.810	1.875	22.072	1	0.000	6 703.704
Extracapsular LNs involving the esophagus or not	3.506	0.869	16.286	1	0.000	33.320
T4 stage or not	2.257	0.810	7.768	1	0.005	9.558
BED_−10_	−0.085	0.023	13.206	1	0.000	0.918

## DISCUSSION

RT plays an important role in the treatment of esophageal carcinoma. Studies focusing on the esophageal response to radiation have increased lately, but all of them have examined non-esophageal cancer [7–9], and data on injury from esophageal cancer, especially esophageal perforation is rare.

We retrospectively analyzed the data of 322 patients with esophageal cancer and found that 18 patients had esophageal perforation during or after RT, for an incidence rate of 5.6%. Perforation due to tumor progression was excluded by therapeutic evaluation. Age less than 60, T4 stage, extracapsular LNs involving the esophagus, re-irradiation and higher BED_−10_ were found to be risk factors for perforation during or after RT.

People with extracapsular LNs involving the esophagus were found to be at higher risk of esophageal perforation, which has not been reported till now. ECLNI confirmed by pathology was a negative prognostic factor for esophageal carcinoma, and patients with ECLNI had a shorter survival time [10–13]. Theoretically, if the LN was large and had necrosis inside the lesion, this may have adverse effects on radiosensitivity. In practice, however, LN volume shrinkage of > 50% was observed in seven of the 12 patients in the perforated group. Once the LNs invaded the esophagus from the tunica adventitia to the tunica intima, a fistula may have formed after necrosis of the tumor from the esophagus throughout the LN. It is important to pay special attention to the esophagus invaded by extracapsular LN, especially for those tumors with a good response. Young patients were found to have a higher risk of perforation. This may be due to the high proportion of concurrent chemotherapy in this group, which may accelerate the shrinkage of the tumor but suppress the repair of normal tissue.

T4 stage was another risk factor, mainly because the lesion has already invaded adjacent structures. When a tumor shrinks during therapy, a fistula may form inside the lesion, which may connect the esophagus to the trachea or bronchus via an esophageal–branchial fistula, to the aorta as an esophageal–aortic fistula, or to the mediastinum as an esophageal–mediastinal fistula. In the study of concurrent CRT for locally advanced esophageal cancer by Atsushi Ohtsu *et al*. [14], five of 54 patients developed a fistula during treatment, but no perforations occurred in patients with non-T4 disease. In our study, 12 of the 18 patients with perforation had T4 disease, and another five patients had extracapsular LNs that invaded the adjacent esophagus. These LNs may play an important role in the formation of perforation, as discussed above, and may produce similar clinical conditions to those of T4 stage. For patients with T4 stage or extracapsular LNs, it may be important to regularly have thoracic CT or barium meal examination to rule out niches during RT, for example, when the total dose reaches 30 or 40 Gy, especially for those with quick alleviation of symptoms.

It was not known whether re-irradiation definitely increased the risk of esophageal perforation. The treatment options after local recurrence includes surgery, chemotherapy and RT [15–17], and data about patients receiving re-irradiation is rare [18]. For various reasons, patients with local recurrence in our study did not receive surgery. After careful consideration about the tolerance of normal tissue, and discussion with the patients, re-irradiation was given to 12 patients. The total mean dose for them was 126.9 Gy (BED_−10_); nine patients had complete response or partial response, and the other three had stable disease after re-irradiation. It seemed that re-irradiation helped control the local disease in some of the patients, even achieving complete response. Previous irradiation was not an absolute contraindication, but the risk of perforation was much higher; multivariate analysis revealed that these patients had increased risk of esophageal perforation after re-irradiation. Considering re-irradiation was the only choice for some patients, and that it may have achieved local control, it appears advisable that some patients should receive a second cycle of RT. One has to weigh up the possibility of perforation; for those with T4 disease or extracapsular LNs invading the esophagus, the risk was found to be much higher. Though we cannot give the precise dose here, we suggest the total dose be as low as possible, as higher doses increase the risk of perforation.

The treatment after esophageal perforation is divided into treatment for perforation and related complications, and treatment for cancer. The principles of treatment for perforation are to control the infection, to prevent continuing septic contamination, to provide nutritional support, and to restore digestive tract continuity. Restoration of digestive continuity includes treatments such as esophagectomy, endoscopic clipping, placement of a self-expandable metallic stent, or T drainage [19]. Esophagectomy is impossible for most patients after perforation from esophageal carcinoma; instead, placement of a self-expandable metallic stent is now used more and more frequently [20–21] as it helps to seal the perforation and avoid the aggravation of infection. After placement of a stent, 11 patients in our study could eat food.

Though effective in the restoration of digestive continuity, several complications were reported after the placement of a stent, and some of them were lethal, including migration of stent, esophagus–trachea fistula, and massive hemorrhage [22–24]. In a study from Lu *et al*. [22], fatal bleeding developed in 6.9% (15/216) of patients. These patients developed massive hematemesis and died shortly thereafter. Massive hemorrhage occurred in three patients after placement of a stent in this study, and another patient developed a new perforation at the edge of the stent. It was difficult to establish causality between the stent and the subsequent hemorrhage and perforation, but it is clear that close follow-up for severe adverse effects after stenting is necessary.

Another difficult decision in treatment after perforation is whether or not to continue antitumor treatment. There are several studies that suggest continuation of CRT after perforation [25–26]. In a study from Muto *et al*. [25], closure of a fistula after CRT was observed in 11 of 12 patients, and the median overall survival was 198 days. They proposed that the presence of a malignant fistula did not contraindicate CRT, and that once inflammation due to the fistula had been controlled, CRT should be utilized because it may provide the best chance for survival and palliation of severe dysphagia. In a study from Koike *et al*. [26], 16 patients with fistulous esophageal carcinoma were treated with CRT, and seven of them developed a fistula during CRT. Treatment in their study consisted of 60 Gy in 30 fractions combined with two cycle of chemotherapy. Disappearance of a fistula was noted during or after CRT in seven patients, and the median survival was 8.5 months. Conflicting with these reports, the two patients in our study who continued RT after stenting and control of infection did not benefit. After the continuation of treatment, one had a new perforation, the other had a massive hemorrhage, and both died quickly. The new perforation and the hemorrhage may have been related to the continuing of RT, the tumor itself, the stent, or even the poor general condition, but it was hard to confirm the cause. It remains difficult to conclude whether or not to continue anticancer treatment. It seems more reasonable to make a decision according to the individual situation.

The median survival of patients with a perforation was 2 months in our study, which was much shorter than that of the non-perforation group, and this is similar to other data [27]. It was demonstrated that prognosis is poor once perforation has occurred, and protection of the esophagus from perforation is thus very important.

This study had some limitations, being a retrospective analysis with treatment delivered differing between patients. The sample of patients with perforation in this study was small, and this may have influenced the statistical analysis. Several years may be required before sufficient cases can be gathered for further analysis, as this complication after RT is rare.

## CONCLUSION

In conclusion, prognosis after perforation was poor. Patients with T4 stage disease, extracapsular LNs involving the esophagus, and those receiving a second course of RT should be given particular care to avoid perforation.
